# ﻿*Spiradiclisscorpiura* (Rubiaceae), a new species from Guangxi, China

**DOI:** 10.3897/phytokeys.252.139783

**Published:** 2025-02-10

**Authors:** You Nong, Li-Qun Lei, Lei Wu, Qi-Min Hu, Ying-Jing Li, Xin-Cheng Qu, Chuan-Gui Xu, Gui-Yuan Wei

**Affiliations:** 1 Guangxi Key Laboratory of Traditional Chinese Medicine Quality Standards, Guangxi Institute of Chinese Medicine & Pharmaceutical Science, No. 20–1 Dongge Road, Nanning, Guangxi, China Guangxi Institute of Chinese Medicine & Pharmaceutical Science Nanning China; 2 Nanning Botanical Garden, Nanning Qingxiushan Scenic and Historic Tourism Development Co., Ltd, Nanning, Guangxi, China Nanning Botanical Garden; Nanning Qingxiushan Scenic and Historic Tourism De-velopment Co., Ltd Nanning China; 3 College of Forestry, Central South University of Forestry and Technology, Changsha, Hunan, China Central South University of Forestry and Technology Changsha China

**Keywords:** Daxin County, limestone, new species, sinkhole, taxonomy

## Abstract

*Spiradiclisscorpiura* (Rubiaceae), a new calcareous species found in Guangxi, China, is described and illustrated. This new species is similar to *S.coccinea*, *S.scabrida*, and *S.purpureocaerulea* in having pubescent stems and subglobose capsules, but it is easily distinguished by its cincinnous inflorescence and its triangular, 1–2 mm long, pubescent bracteoles. According to the IUCN criteria, it is considered Data Deficient (DD) until more information becomes available. Photographs, an illustration, a distribution map, and a comparative table with the most similar species are provided.

## ﻿Introduction

*Spiradiclis* Blume closely resembles *Ophiorrhiza* L., and the two genera are in the tribe Ophiorrhizeae based on morphological characteristics (Verdcourt 1958; Darwin 1976; Lo 1999; Chen and Taylor 2011; Wu et al. 2019) and molecular evidence (Bremer 2009; Rydin et al. 2009; Wikström et al. 2013; Razafimandimbison and Rydin 2019). Razafimandimbison and Rydin (2019) suggested that *Spiradiclis* is a synonym of *Ophiorrhiza*. However, we consider that the delimitation and relationship of the two genera still need further research, and since *Spiradiclis* is morphologically different from *Ophiorrhiza* by its linear-oblong or subglobose capsules with four valves (vs. obcordate and compressed capsules with two valves), we prefer the traditional concept of *Spiradiclis*, thereby considering it separate from *Ophiorrhiza*.

There are a total of 62 *Spiradiclis* species, according to Plants of the World Online (POWO 2024). They are distributed in southeastern Asia, including Bhutan, China, India, Indonesia, Myanmar, and Vietnam. Most of the species are distributed in China and are native to the south and southwest of the country. In the last decade, more than 20 new species of *Spiradiclis* have been discovered in China (e.g., Wang 2016; Zhang et al. 2018; Pan et al. 2019; Tong et al. 2020; Cai et al. 2022; Nong et al. 2024).

During field surveys in Daxin County, Guangxi, in July 2024, a *Spiradiclis* population was found in flower and fruit that was morphologically similar to *Spiradicliscoccinea* H.S.Lo. However, this newly collected *Spiradiclis* is distinctly different from *S.coccinea* by its elliptic leaves and cincinnous inflorescence. Therefore, this population was suspected to represent a new species. This was confirmed by more observations, the examination of specimens of closely related *Spiradiclis* species from the herbaria PE, IBK, GXMI, and KUN, and by consulting relevant literature. Hence, we confirm that the unusual plant is a species of *Spiradiclis* new to science, and the newly discovered taxon is here described as a new species.

## ﻿Materials and methods

Fieldwork was carried out in Daxin County, Guangxi, to document the new species in its natural habitat. In addition, studies of herbarium material of various *Spiradiclis* species were conducted at PE, IBK, GXMI, and KUN, and relevant literature was consulted (Lo et al. 1983; Wang 2002; Wang et al. 2015; Wu et al. 2015, 2016, 2019; Pan et al. 2016; Liu et al. 2017; Zhang et al. 2018; Wen et al. 2019; Li et al. 2021; Song et al. 2022). Additional related *Spiradiclis* species were examined based on online images from the Kew Herbarium Catalogue (http://apps.kew.org/herbcat/gotoHomePage.do) and JSTOR Global Plants (http://plants.jstor.org/). Morphological characteristics of stems, leaves, pedicels, flowers, receptacles, gynoecia, and carpels were used to distinguish *Spiradiclis* species in this study.

The description is based on the type specimens. Measurements were made with a tape measure and callipers. The structure of the indumentum and its distribution were observed and described using a dissecting microscope at magnifications of more than 20×. Additional information on locality, habitat, ecology, plant form, and fruits was collected in the field. The preliminary conservation threat assessment followed IUCN Categories and Criteria (IUCN 2022).

## ﻿Results and discussion

### ﻿Taxonomy

#### ﻿ Spiradiclis scorpiura

Taxon classificationPlantaeGentianalesRubiaceae

Y.Nong & L.Wu sp. nov.

029F4BF4-FE9B-555A-8239-DD1D39A807AB

urn:lsid:ipni.org:names:77356517-1

Figs 1 –4

##### Chinese name.

xiē wěi luó xù cǎo (蝎尾螺序草).

##### Diagnosis.

*Spiradiclisscorpiura* is most similar to *S.coccinea* but is different in its densely pubescent young stems that become glabrous when older (vs. glabrous or subglabrous), its cincinnous inflorescences (vs. cymose), its triangular, 1–2 mm long, pubescent bracteoles (vs. subulate, 3–4 mm long, glabrous), its calyx puberulent outside (vs. glabrescent outside), and its capsule 3–4 mm in diam. (vs. 4.5–5.5 mm in diam.).

**Figure 1.**
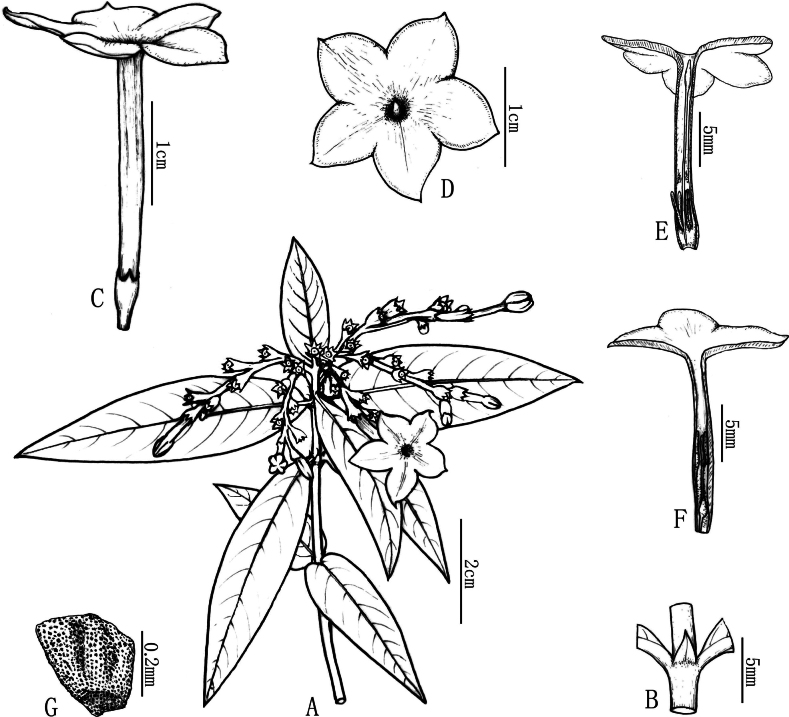
Line drawing of *Spiradiclisscorpiura* Y.Nong & L.Wu **A** flowering plant **B** stipule **C** flower **D** long-styled flower, frontal view, showing corolla lobes and stigma **E** longitudinally opened long-styled flower, showing the position of the stamens and the style and stigma **F** longitudinally opened short-styled flower, showing the position of the stamens and the style and stigma **G** seed (Drawn by Xin-cheng Qu).

##### Type.

China • Guangxi, Daxin County, 22°54'06"N, 106°50'02"E, alt. 504 m, at the rim of the top of a sinkhole, 11 July 2024, flowering, *Y. Nong NY2024071101* (GXMI). (***Holotype***: GXMI! 051187; isotype: IBK!).

##### Description.

Subshrubs, perennial, 20–50 cm tall, rooting near base, stems ascending; stems densely pubescent when young but glabrous when old. Leaves opposite; petiole 1–2 mm long, sparsely pubescent; blade drying papery, adaxially olive green, abaxially yellowish green, elliptic, 3–7 × 0.5–1.5 cm, sparsely pubescent or glabrous on both surfaces, margin entire, base cuneate, apex acuminate; secondary veins 8–12 on each side of the midrib, midrib concave adaxially and prominently convex abaxially; stipules triangular, 1–2 mm long, glabrous outside, apex acute. Inflorescences terminal, cincinnous, 3–6 branched, 3–44-flowered, pubescent; peduncles 0.6–1 cm long, pubescent; pedicels short, c. 1 mm long, pubescent; bracteoles triangular, 1–2 mm long, pubescent outside. Flowers distylous. Calyx pubescent; hypanthium portion obovate, 1–2 mm long, with 5 straight ridges; lobes 5, triangular or ovate-lanceolate, 1–1.5 mm long. Corolla purple, slenderly salverform-funnelform, glabrous or pubescent outside; tube 15–18 mm long, lobes broadly ovate to suborbicular, 6–8 mm long. Stamens 5. Style filiform, stigma clavate, 2-lobed, lobes linear, c. 2 mm long. Long-styled flowers: corolla tube with pilose ring above stamens inside; stamens born near the base of the tube, anthers sessile or subsessile, c. 3 mm long; style c. 1.5 cm long or slightly longer. Short-styled flowers: corolla tube pubescent near the base inside; stamens born in the middle of the tube, anthers sessile or subsessile, c. 2 mm long; style c. 4 mm long. Capsules subglobose, 3–4 mm in diam., glabrescent, valves 4. Seeds numerous, angular, c. 0.2 mm in diam.

**Figure 2.**
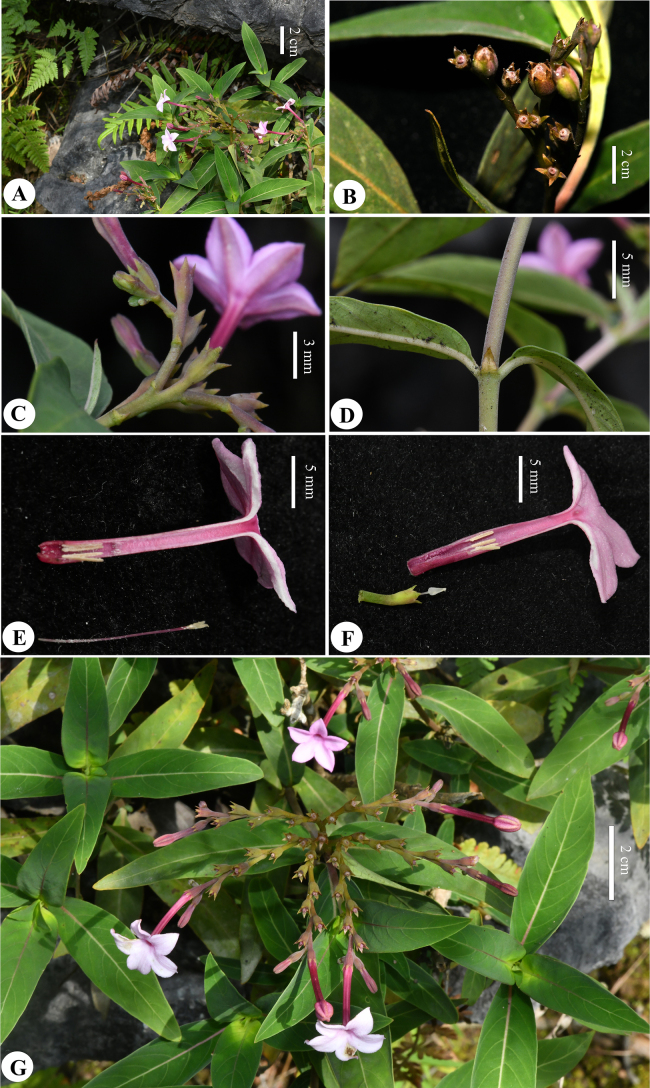
*Spiradiclisscorpiura* Y.Nong & L.Wu **A** habit **B** detail of fruiting plant **C** inflorescence **D** stipule **E** longitudinally opened, long-styled flower **F** longitudinally opened, short-styled flower **G** flowering plant (photographed and edited by You Nong).

##### Phenology.

Flowering and fruiting in June–July.

##### Etymology.

The specific epithet “*scorpiura*” refers to the terminal, cincinnous inflorescences of the new species.

**Figure 3.**
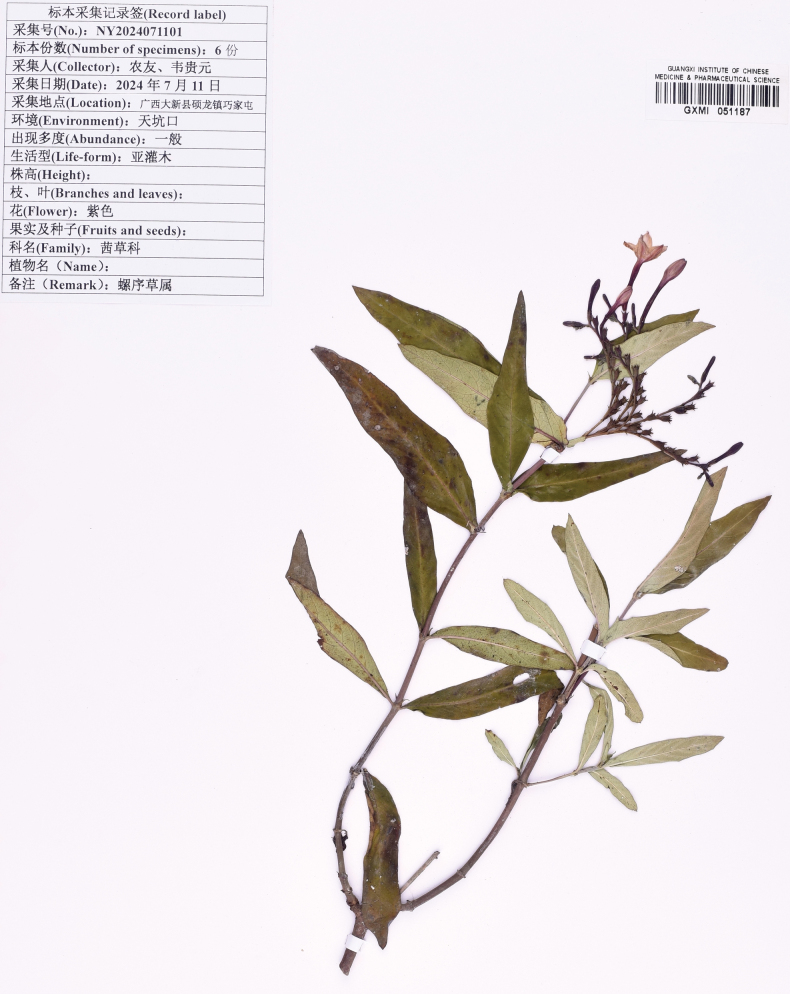
Holotype specimen of *Spiradiclisscorpiura* Y.Nong & L.Wu, *Y. Nong NY2024071101* (GXMI! 051187).

##### Distribution and habit.

Known only from southeast Guangxi, China. The species has only been found at the rim of a sinkhole at elevations of 504 m.

##### Preliminary IUCN red list category.

Data available for the new species, only known from the type locality and the type specimens, are insufficient to assess its conservation status. According to the IUCN Criteria (IUCN 2022), it is considered Data Deficient (DD) until more information becomes available. *Spiradiclisscorpiura* is currently known from a single, relatively large population. Further collection and monitoring are necessary to allow more conclusive estimations about the rarity and vulnerability of the species.

**Figure 4.**
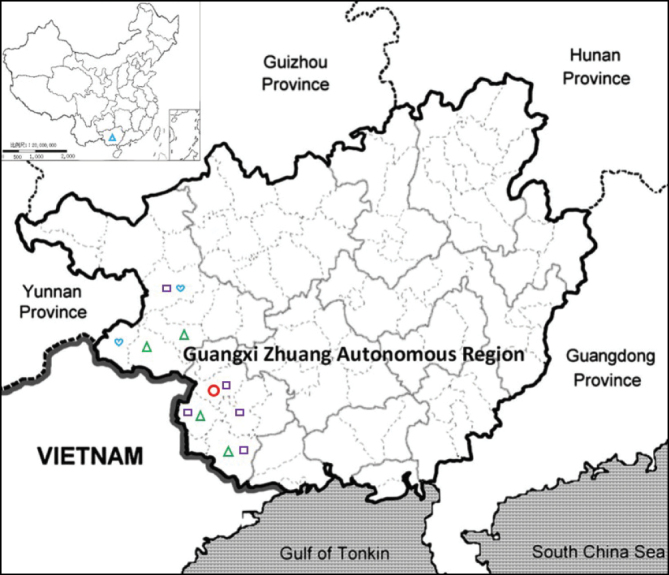
Distribution of *Spiradiclisscorpiura* (red circle), *S.coccinea* (green triangle), *S.scabrida* (blue heart), and *S.purpureocaerulea* (purple square) in Guangxi (blue triangle in insert map), China.

### ﻿Comparison with other *Spiradiclis* species

In addition, *S.scorpiura* also looks similar to *S.scabrida* D.Fang & D.H.Qin, but is different by its shorter petioles (1–2 mm vs. 2–5 mm long), its cincinnous inflorescences (vs. corymbose-cymose), and its triangular, 1–2 mm long, pubescent bracteoles (vs. linear, 2–5 mm long, glabrous). *Spiradiclisscorpiura* is also similar to *S.purpureocaerulea* H.S.Lo, but it differs in its densely pubescent young stems that become glabrous when old (vs. densely pubescent young and old stems), its elliptic leaves, pubescent or glabrous on both surfaces (vs. ovate, adaxially densely strigose-hispidulous, abaxially densely pubescent), its 1–2 mm long petioles (vs. 5–20 mm long), its cincinnous inflorescences (vs. congested-cymose), and its triangular, 1–2 mm long, pubescent bracteoles (vs. narrowly lanceolate, 4–5 mm long, densely pubescent). More detailed morphological differences amongst the similar species are shown in Table 1.

**Table 1.** Main morphological differences between *Spiradiclisscorpiura*, *S.coccinea, S.scabrida*, and *S.purpureocaerulea*.

Morphological traits	*S.scorpiura*	*S.coccinea*	*S.scabrida*	*S.purpureocaerulea*
**Stems**	densely pubescent when young but glabrous when old	glabrous or subglabrous	pubescent to glabrescent	densely pubescent
**Leaves**	elliptic, sparsely pubescent or glabrous on both surfaces	narrowly elliptic-oblong or elliptic-oblong, glabrous on both surfaces	ovate, narrowly ovate, or lanceolate, abaxially glabrous or occasionally sparsely strigillose or scabridulous at least on principal veins	ovate, adaxially densely strigose-hispidulous, abaxially densely pubescent
**Length of petioles**	1–2 mm	1–2 mm	2–5 mm	5–20 mm
**Stipules**	triangular, 1–2 mm long, glabrous outside	triangular, rapidly narrowed to subulate, 4–5 mm long, glabrous outside	subtriangular, 0.7–1 mm long, subglabrous outside	subulate, 2–3 mm long, pubescent outside
**Inflorescence**	cincinnous, 3–6-branched, pubescent	cymose, with more than 10 flowers, pubescent	corymbose-cymose, 3-24-flowered, pubescent, puberulent, or glabrescent	congested-cymose, densely pubescent
**Bracteoles**	triangular, 1–2 mm long, pubescent outside	subulate, 3–4 mm long, glabrous outside	linear, 2–5 mm long, glabrous outside	narrowly lanceolate, 4–5 mm long, densely pubescent outside
**Calyx**	puberulent outside; hypanthium portion obovate, 1–2 mm long, with 5 straight ridges; lobes triangular or ovate-lanceolate, 1–1.5 mm long	glabrescent outside; hypanthium portion obconic, 1.2–1.5 mm long; lobes narrowly lanceolate, 1.7–2 mm long	puberulent outside; hypanthium portion obovate, 1–1.5 mm long; lobes ovate-lanceolate, 1–1.5 mm long	pubescent outside; hypanthium portion obconic-globose, c. 2 mm long; lobes narrowly lanceolate, 4–4.5 mm long
**Corolla**	tube 15–20 mm long, lobes broadly ovate to suborbicular, 6–8 mm long	tube 15–18 mm long, lobes broadly ovate to suborbicular, 4.5–6 mm long	tube 25–26 mm long; lobes ovate, c. 3.5 mm long	tube 19–21 mm long; lobes subovate, c. 6 mm long
**Capsule**	subglobose, 3–4 mm in diam.	subglobose, 4.5–5.5 mm in diam.	subglobose, 3–4 mm in diam.	subglobose, 4–4.5 mm in diam.

## Supplementary Material

XML Treatment for Spiradiclis scorpiura

## ﻿Additional information

### Author ORCIDs

You Nong https://orcid.org/0000-0001-7004-0946

Li-Qun Lei https://orcid.org/0009-0009-7319-5439

Lei Wu https://orcid.org/0000-0003-1451-7855

Qi-Min Hu https://orcid.org/0009-0003-0490-3557

Ying-Jing Li https://orcid.org/0009-0008-4849-2426

Xin-Cheng Qu https://orcid.org/0009-0009-9078-9976

Chuan-Gui Xu https://orcid.org/0009-0000-6263-3821

Gui-Yuan Wei https://orcid.org/0000-0003-0652-1213

### Data availability

All of the data that support the findings of this study are available in the main text.

## References

[B1] Bremer B (2009) A review of molecular phylogenetic studies of Rubiaceae. Annals of the Missouri Botanical Garden 96(1): 4–26. 10.3417/2006197

[B2] Cai JH Shui YM Song XF Wu L (2022) Validation of the name *Spiradicliselliptica* (Rubiaceae), a new species endemic to southwestern China. Phytotaxa 545(1): 110–114. 10.11646/phytotaxa.545.1.10

[B3] Chen T Taylor CM (2011) *Spiradiclis*. In: Wu ZY Raven PH Hong DY (Eds) Flora of China.Vol. 19. Science Press, Beijing & Missouri Botanical Garden Press, St. Louis, 330–339.

[B4] Darwin SP (1976) The Pacific species of *Ophiorrhiza* L. (Rubiaceae). Lyonia 1: 48–101.

[B5] IUCN (2022) Guidelines for using the IUCN Red List Categories and Criteria, version 14. Prepared by the Standards and Petitions Committee. https://www.iucnredlist.org/resources/redlistguidelines [Accessed 15 October 2024]

[B6] Li JL Yuan Q Liu Y Song XF Pan B Qu CH Wu L (2021) Two new species of *Spiradiclis* (Rubiaceae) from limestone areas in southwestern China. Nordic Journal of Botany 39: e02979. 10.1111/njb.02979

[B7] Liu J Pan B Li SW Xu WB (2017) *Spiradiclisquanzhouensis* (Rubiaceae): A new species from limestone area in Guangxi, China. Nordic Journal of Botany 36(3): e01595. 10.1111/njb.01595

[B8] Lo HS (1999) *Spiradiclis* Blume. In: Lo HS (Ed.) Flora Reipublicae Popularis Sinicae.Vol. 71(1). Science Press, Beijing, 86–110.

[B9] Lo HS Sha WL Chen XX (1983) A revision of the genus *Spiradiclis* Bl. Acta Botanica Austro Sinica 1: 27–36.

[B10] Nong Y Lei LQ Wei GY Qu XC Zhao ZY Feng B Xu CG Wu L (2024) *Spiradiclisyanii* (Rubiaceae), a new species from Guangxi, China. PhytoKeys 247: 173–181. 10.3897/phytokeys.247.123867 39444565 PMC11496824

[B11] Pan B Ma HS Wang RJ (2016) *Spiradiclispengshuiensis* (Ophiorrhizeae, Rubioideae), a new species from Chongqing, China. PhytoKeys 63: 41–45. 10.3897/phytokeys.63.8016 PMC4956927 27489477

[B12] Pan B Tu RH Hareesh VS Wu L (2019) *Spiradicliscavicola* (Rubiaceae), a new species from limestone caves in south-western China. Annales Botanici Fennici 56(1–3): 1–4. 10.5735/085.056.0101

[B13] POWO (2024) Plants of the World Online. Facilitated by the Royal Botanic Gardens, Kew. https://powo.science.kew.org/results?f=&q=Spiradiclis [Accessed 18 October 2024]

[B14] Razafimandimbison SG Rydin C (2019) Molecular-based assessments of tribal and generic limits and relationships in Rubiaceae (Gentianales): Polyphyly of Pomazoteae and paraphyly of Ophiorrhizeae and *Ophiorrhiza.* Taxon 68(1): 72–79. 10.1002/tax.12023

[B15] Rydin C Kainulainen K Razafimandimbison SG Smedmark JEE Bremer B (2009) Deep divergences in the coffee family and the systematic position of *Acranthera*. Plant Systematics and Evolution 278(1–2): 101–123. 10.1007/s00606-008-0138-4

[B16] Song XF Liu WJ Chen AX Yao ZM Lan HB Wu L (2022) *Spiradiclisliboensis* (Rubiaceae), a new species from limestone mountain areas in Guizhou, China. PhytoKeys 204: 73–81. 10.3897/phytokeys.204.84397 36760616 PMC9848952

[B17] Tong YH Xia NH Wu L Vu TC (2020) Critical notes on *Spiradiclispurpureocaerulea* H.S. Lo (Rubiaceae) from Vietnam. Adansonia 42(19): 291–296. 10.5252/adansonia2020v42a19

[B18] Verdcourt B (1958) Remarks on the classification of the Rubiaceae. Bulletin du Jardin botanique de l’État à Bruxelles 28: 209–281.

[B19] Wang RJ (2002) Two new species of *Spiradiclis* (Rubiaceae) from China. Novon 12(3): 420–423. 10.2307/3393092

[B20] Wang RJ (2016) *Spiradiclisjingxiensis* sp. nov. (Rubiaceae) from Guangxi, China. Nordic Journal of Botany 34(5): 550–552. 10.1111/njb.01134

[B21] Wang RJ Wen HZ Deng SJ Zhou LX (2015) *Spiradiclisdanxiashanensis* (Rubiaceae), a new species from south China. Phytotaxa 206(1): 30–36. 10.11646/phytotaxa.206.1.5

[B22] Wen ZJ Yang JC Xu YF Wu L (2019) *Spiradiclisdensa* sp. nov. (Rubiaceae) from limestone areas in Guangxi, China. Nordic Journal of Botany 37(6): e02190. 10.1111/njb.02190

[B23] Wikström N Neupane S Kårehed J Motley TJ Bremer B (2013) Phylogeny of *Hedyotis* L. (Rubiaceae: Spermacoceae): redefining a complex Asian-Pacific assemblage. Taxon 62(2): 357–374. 10.12705/622.2

[B24] Wu L Wang JL Liu QR (2015) *Spiradiclispauciflora* (Rubiaceae), a new species from limestone areas in Guangxi, China. Annales Botanici Fennici 52(3–4): 257–261. 10.5735/085.052.0318

[B25] Wu L Tong Y Pan B Liu QR (2016) *Spiradiclisglabra* sp. nov. (Rubiaceae) from limestone areas in Guangdong, China. Nordic Journal of Botany 34(6): 718–721. 10.1111/njb.01156

[B26] Wu L Wang BM Pan B Yu XL (2019) *Spiradiclistubiflora* (Rubiaceae), a new cave-dwelling species from southern China. PhytoKeys 130: 217–224. 10.3897/phytokeys.130.34625 31534408 PMC6728394

[B27] Zhang F Liu Y Wen ZJ Wu L (2018) *Spiradiclislui*, a new species of Rubiaceae from Guangxi, China. Nordic Journal of Botany 36(6): e01786. 10.1111/njb.01786

